# Exploring violence towards EMS personnel: a multiprofessional qualitative study

**DOI:** 10.1186/s12873-026-01530-x

**Published:** 2026-03-16

**Authors:** Heikki Riihimäki, Laura-Maria Peltonen, Mari Lahti, Jani Paulin

**Affiliations:** 1 Turku University of Applied Sciences and Turku University Hospital, Turku, Finland; 2 University of Eastern Finland, Kuopio University Hospital, Turku University Hospital, Turku, Finland; 3 Turku University of Applied Sciences and University of Turku, Turku, Finland

**Keywords:** Prehospital emergency care, Emergency medical services, Violence, Occupational safety, Patient aggression, Occupational health

## Abstract

**Background:**

Violence against emergency medical service personnel is common and often underreported, with verbal abuse being the most frequent form, but physical and sexual violence also occur. The consequences of such violence range from psychological distress to physical harm and organizational burden. Despite growing evidence of the phenomenon, the dynamic, situational and interactional nature of this violence as well as effective prevention strategies are still insufficiently understood. The objective of this study was to examine factors contributing to violence against EMS personnel before, during and after violent incidents.

**Methods:**

This qualitative study employed two multiprofessional stakeholder workshops (*n* = 36) conducted in Finland in October 2024. Participants included professionals from the EMS setting and its related fields, as well as patient representatives. The workshops produced experiences and perspectives on violence against EMS personnel, focusing on incident dynamics of violent encounters. Data was analyzed using thematic analysis.

**Results:**

Violence towards EMS personnel presents itself through three interrelated themes: the aggressor, the EMS personnel, and the organization and the EMS system. The findings indicate that these themes interact dynamically, with violent incidents shaped by individual behavior, professional practice and institutional conditions. Aggressor-related factors such as substance use, psychiatric illness, and altered mental states were described as increasing risk for violence. EMS personnel’s fatigue, inexperience, and moralizing attitudes were seen to increase vulnerability, whereas resilience and situational awareness were protective. At the organizational level, unclear risk-assessment protocols, inconsistent cooperation with the police, and limited post-incident support emerged as key challenges.

**Conclusions:**

Violence against EMS presents itself as a multifactorial and dynamic phenomenon involving aggressor behavior, EMS personnel actions and organizational structures. Embedding de-escalation and disengagement competencies into training, strengthening system-level safeguards and implementing post-incident support are essential. The development of predictive models on how to manage risks at every stage of the EMS mission is warranted.

**Supplementary Information:**

The online version contains supplementary material available at 10.1186/s12873-026-01530-x.

## Background

Violence towards emergency medical services (EMS) personnel has been identified as a common and growing phenomenon in the prehospital setting [1, 2]. Over two-thirds of EMS personnel report experiencing some violence annually during EMS missions [1, 3]. Studies have reported an incidence rate of violence in 0.7%–1,16% of all EMS missions [4–6]. Underreporting has been identified as a major factor and the actual prevalence is most likely higher [2, 6–8]. The rates of different types of violence vary but verbal violence is the most common [1, 3, 4, 9]. Most reported violent incidents (57,2–62,3%) are verbal, while 16,8–24,2% are physical. However, in 13,1–26,0% of cases, both forms of violence co-occur [4, 6]. Sexual violence and harassment have also been reported to be common in EMS settings [10]. Violence has severe consequences for EMS personnel’s safety and wellbeing, ranging from emotional and psychological stress to physical injuries followed by a possible loss of work time and increased system costs [2, 7, 9, 11].

Several patient-related risk factors for violence against EMS personnel have been identified, including male sex [3, 4, 6], younger age [4–6], alcohol and drug abuse [1, 3, 4, 6–9], altered mental state [1, 3, 5–7, 9], urban working environment [1, 4, 9], night time [4, 6, 7] and higher National Early Warning Score (NEWS) [4]. The overwhelming majority of violent incidents have been attributed to patients, whereas a smaller proportion have involved family members or bystanders [1, 3, 4, 7–9]. Colleague-to-colleague violence has been recognized in workplace settings, but research within prehospital EMS appears limited [9]. Risk factors related to the EMS personnel include the type of EMS unit, personnel composition and level of experience. Advanced Life Support (ALS) units tend to face higher rates of violent incidents than Basic Life Support (BLS) units [4]. Paramedics report experiencing more violence compared to emergency medical technicians and firefighters [1, 2, 7]. In addition, inexperienced and younger EMS personnel are at an increased risk of violent encounters [1, 3, 8]. Research into EMS personnel’s gender as a risk factor has been inconclusive [1, 3, 7–9].

While prior work has examined prevalence and risk factors associated with violence towards EMS personnel, the dynamic, situational and interactional nature of this violence remains insufficiently understood. There is also a lack of validated strategies for violence prevention and management [11]. Hence, a more nuanced investigation of the underlying mechanisms and situational triggers of violent incidents in EMS settings is still needed. The objective of this study was to examine factors contributing to violence against EMS personnel before, during and after violent incidents.

## Methods

### Study design

This study utilized a qualitative exploratory research design incorporating stakeholder workshops. Structured stakeholder workshops served as the data collection method, engaging participants (*n* = 36) from diverse professional backgrounds. Workshops were carefully designed to facilitate open dialogue and collaborative reflection, enabling participants to share perceptions and possible experiences of violence within EMS settings. Data gathered during these interactive sessions were analyzed using thematic analysis.

### Setting

The Finnish EMS provides prehospital emergency care and patient conveyance when needed, operating under the Wellbeing Services Counties responsible for population health service [12]. Integrated into the national emergency response network, the EMS works closely together with the emergency response centres (ERC), rescue services, police, and healthcare institutions. ERCs use a standardized protocol to determine call priority, assign resources and guide callers [13, 14]. EMS staffing and treatment capabilities vary by region and call-priority, including EMTs, firefighters, paramedics, field supervisors and emergency physicians.

### Data collection

Data collection occurred in October 2024 through two 1-day multiprofessional stakeholder workshops. Both workshops featured a similar composition of participants, representing diverse fields within EMS and related professional groups. Information about the participants is presented in Table 1.

**Table 1:** Workshop participant information

Occupation / Role	Participants (*n*)	Work experience (years)	Sex (f=female, m=male)	Received additional occupational violence training
Paramedic	11	3–22	f (27%), m (73%)	64%
ERC Specialist	2	11, 21	f (100%), m (0%)	0%
Police officer	2	4, 4	f (50%), m (50%	100%
Paramedic student	3	2, 4, 4 (as EMT)	f (100%), m (0%)	0%
EMS Physician	2	10, 24	f (0%), m (100%)	100%
Occupational specialist	3	4, 21, 40	f (67%), m (33%)	100%
Patient representative (expert by experience)	2	8, 15	f (50%), m (50%)	0%
EMT / Firefighter	6	2–8	f (17%), m (83%)	50%
Occupational health psychologist	1	17	f (100%), m (0%)	100%
EMS Supervisor	2	23, 27	f (50%), m (50%)	100%
EMS Researcher	1	22	f (100%), m (0%)	0%
EMS teacher	1	25	f (0%), m (100%)	100%
**Total**	**36**	**2–40**	**f (44%)**, **m (56%)**	**58%**

Participants were identified and contacted by email through cross-organizational mapping and through existing professional networks. Purposive sampling was used based on the recruits’ professional background, experience and willingness to participate. Goal was to recruit a diverse group of participants with experience and knowledge about the EMS. Patient representatives were recruited through a patient advocacy group. Recruited patient representatives had personal experiences of violent behavior towards EMS personnel in addition to having expert by experience -training. All participants were informed about the study, their rights, including the option to withdraw participation at any point without any consequence. The workshops were intentionally conducted in separate days to minimize potential cross-group influence and ensure independent data generation. No participants withdrew from the study. Workshops were held in an office setting. Only researchers and participants were present during the data collection.

The data originated from a specific structured group task where participants collaboratively explored the sequential dynamics of violent encounters experienced by EMS professionals. Researchers (HR, JP) supervised and facilitated the workshops. Participants on both days were divided into smaller groups of 3 to promote active engagement. Groups were decided by the researchers in a way that every group was as diverse and balanced as possible. Each group had a voluntary participant who would document the group’s discussions and conclusions on a secure digital platform in written form. Participants were instructed to openly discuss and record in their own words the circumstances, experiences and views before, during and after the violent incident during EMS missions. This structured focus on temporal phases was used to capture how different factors emerge across the course of an EMS mission involving violence. No further instructions or restrictions were given to the participants.

### Data analysis

Thematic analysis was adopted as the method for analyzing the collected data. This method was chosen for its methodological flexibility and its capacity to yield rich and detailed insights into a complex phenomenon [15, 16]. The thematic analysis followed Braun and Clarke’s six phases: (1) familiarization with the data, (2) generating initial codes, (3) searching for themes, (4) reviewing themes, (5) defining and naming themes and (6) producing the report [17]. All authors participated in this process. Various themes and sub-themes were inductively identified in the phases of before, during and after a violent incident towards EMS. The resulting themes were revisited multiple times and discussed until consensus was reached about their nature and meaning. Example of the thematic analysis is presented in Table 2.

**Table 2:** Examples of the thematic analysis process

Data extract	Code	Theme	Sub theme
“Social isolation is visible: No work, no income, no place to stay; people end up in situations where violence becomes part of the daily life.”	Social isolation increases the risk of violence. Violence normalized due to hardships.	Aggressor	Social hardships and isolation
“If the communication of the EMS unit doesn’t work, it can make the personnel vulnerable for violence.”	Lack of communication increase the risk of violence.	EMS	Communication with partner

### Rigor and reflexivity

Trustworthiness of the analysis was ensured using the framework of credibility, dependability, confirmability and transferability described by Lincoln and Guba [18]. Credibility was supported by diverse interprofessional participation enabling a broad range of viewpoints. The structure of before-during-after during workshops enhanced the clarity of the temporal factors influencing violent incidents. This allowed participants to articulate specific factors of violence rather than general opinions. Cross-group comparison and data synthesis ensured that the themes reflected shared viewpoints. Dependability was strengthened by applying a standardized workshop structure and a predefined iterative analysis process.

Confirmability was supported by using participants’ own written documentation as the data source, and researchers refrained from influencing the discussions. Any intervening done by the researchers was related to facilitating the workshops. Theme development was grounded in the extracted documentation and reviewed by the authors until consensus was reached to reduce individual interpretation bias. Transferability was strengthened by providing detailed descriptions of the Finnish EMS context, participant information and workshop procedures. Although this study was grounded in a very specific context, the identified mechanisms of violence towards EMS personnel are likely relevant in other similar systems.

The research team included both members with and without professional experience in EMS. The diversity within the team was important and members were already familiar with the phenomenon of violence towards EMS workers and had a strong understanding of EMS work. Their expertise proved strong support in conducting the study and facilitating the workshops while the familiarity with EMS work and professional terminology enhanced the impact of the data analysis.

Participants were recruited through professional networks across various regions in Finland. Some were previously known to the research group, while others were not. To address potential biases and preconceptions, the team engaged in active discussions and reflections. Including researchers with and without an EMS background fostered a range of perspectives and helped prevent the normalization of familiar EMS practices and assumptions.

## Results

**Fig. 1:**
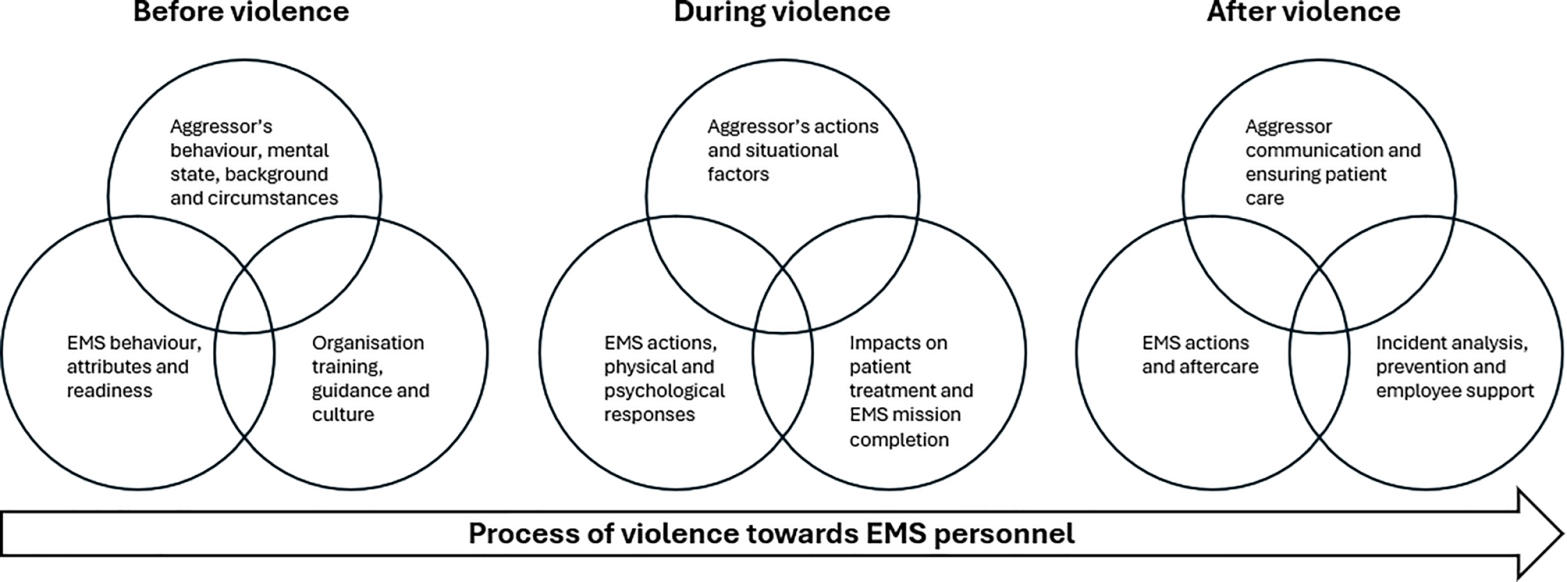
Process of violence towards EMS personnel

The identified main themes were (1) the aggressor, (2) the EMS personnel and (3) the organization and the EMS system, where the latter include factors related to the operative and administrative management of EMS missions and their personnel as well as co-operation with the ERC and other authorities. These themes intersect dynamically, as characteristics of the aggressor, the actions of EMS personnel and organizational or system level factors interact during EMS missions. The analysis resulted in a process model depicting violence towards EMS personnel (Fig. 1). The model illustrates three interrelated phases of before, during and after the violence. In the data, references to the aggressor primarily concerned patients encountered during EMS missions, with occasional mentions of family members or bystanders. There were no accounts involving other sources of violence.

### Before the violent incident

**Fig. 2:**
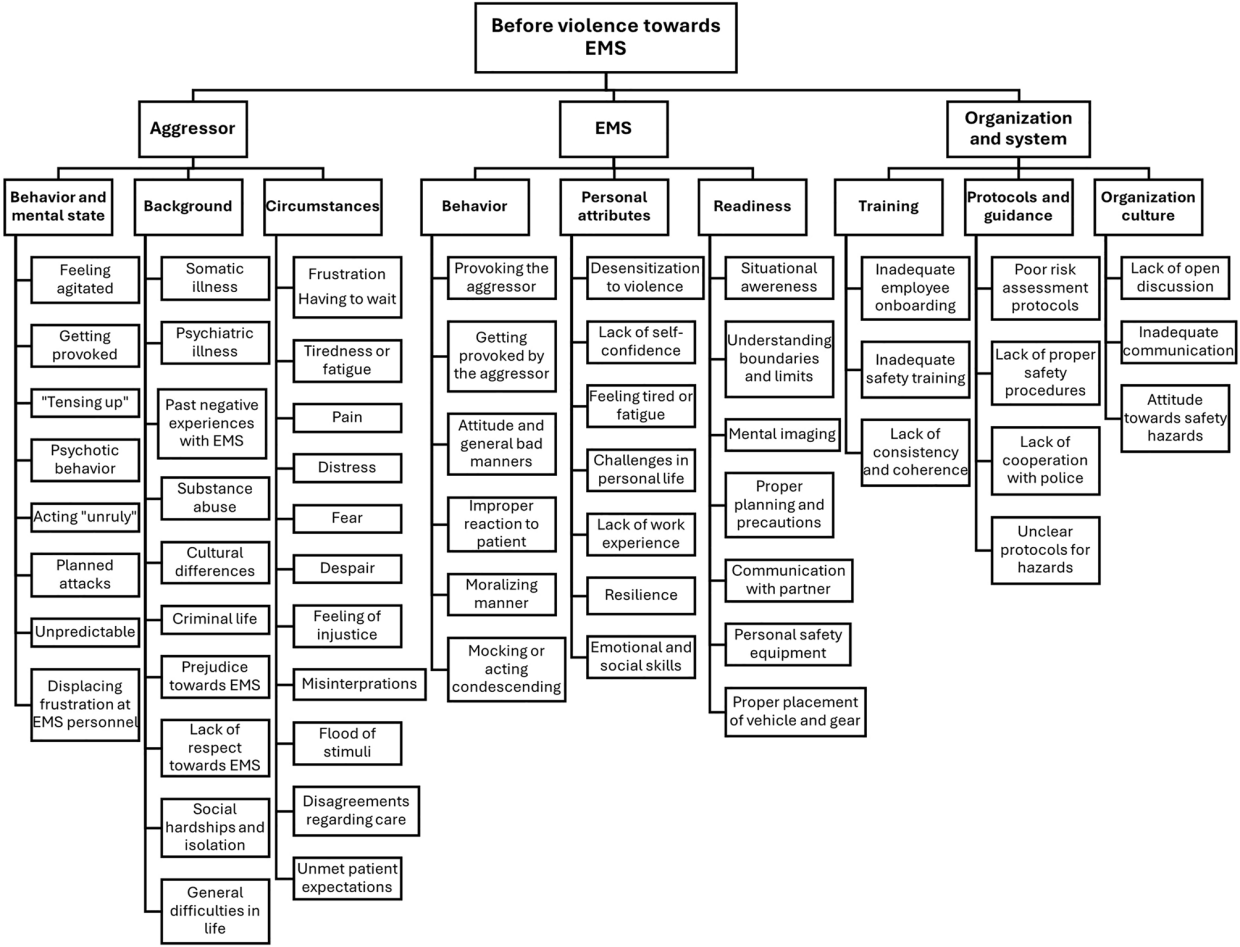
Identified factors preceding violence towards EMS personnel

Before the violence (Fig. 2.), the analysis developed three aggressor-related subthemes capturing behavior, background and circumstances. Aggressors were frequently described as agitated, tense or exhibiting psychotic features. Substance abuse, psychiatric conditions and broader social issues, such as isolation or stress, were often cited as background factors. These were often further affected by circumstantial triggers like fatigue, pain, distress and a sense of injustice. Importantly, EMS interactions could also act as a catalyst for violence: Long wait times, misinterpretation, poor past experiences with EMS or unmet patient expectations were all described as potential triggers. Cultural differences were mentioned as sometimes contributing factors when communicating with an agitated patient or family member, whether it was due to different social dynamics or language barriers.

Signs before violence can include locked-in gaze, body language, tensing up, way they talk or (suddenly) stopped talking. -Workshop 1, group 1a.

Waiting (for EMS) might frustrate the patient. Also pain and distress might promote violent behavior. -Workshop 1, group 1a.

Three EMS-related subthemes were developed capturing personnel behavior, personal attributes and readiness. Interpersonal dynamics were described as vital: Aggression could escalate if EMS personnel respond poorly by provoking, moralizing or condescending. These EMS behavior-related factors were diverse in nature, and they were repeatedly documented during the workshops. Individual EMS vulnerabilities, such as fatigue, low stress tolerance or limited work experience, were also considered as risk factors. Prior exposure to violence could build resilience or foster a false sense of security through desensitization. Participants highlighted emotional and social skills as crucial to defusing tense situations with the aggressor. Readiness was portrayed as involving situational awareness, clear partner communication and proactive safety measures, ranging from mental rehearsal to strategic positioning of the ambulance and equipment to secure rapid disengagement and retreat if needed.

EMS characteristics and social skills make a great difference in how the situation unfolds -Workshop 2, group 1b.

Also, the EMS personnel’s own life has an impact: Time of day, earlier calls, being tired… -Workshop 1, group 1b.

The analysis developed three organizational and systemic subthemes capturing training, protocols and guidance and organization culture. Participants described that onboarding and safety training can be inconsistent, insufficient or not continuously reinforced, leaving EMS personnel unprepared for violent encounters. Gaps in organizational guidance included unclear or missing protocols for risk assessment, limited cooperation with authorities (e.g. the police) and an overall uncertainty over how to act when threats emerged during any stage of the EMS missions. Finally, the organization culture played a critical role: Inadequate communication, lack of open discussion and ununiform attitudes towards occupational safety could create environments where EMS personnel act “as they deem fit” without a shared vision for handling workplace hazards.

Clear protocols and guidelines are needed for the management of violent situations. -Workshop 1, group 1a.

Training has a huge effect. Protocols must be practiced. -Workshop 1, group 2b.

### During the violent incident

**Fig. 3:**
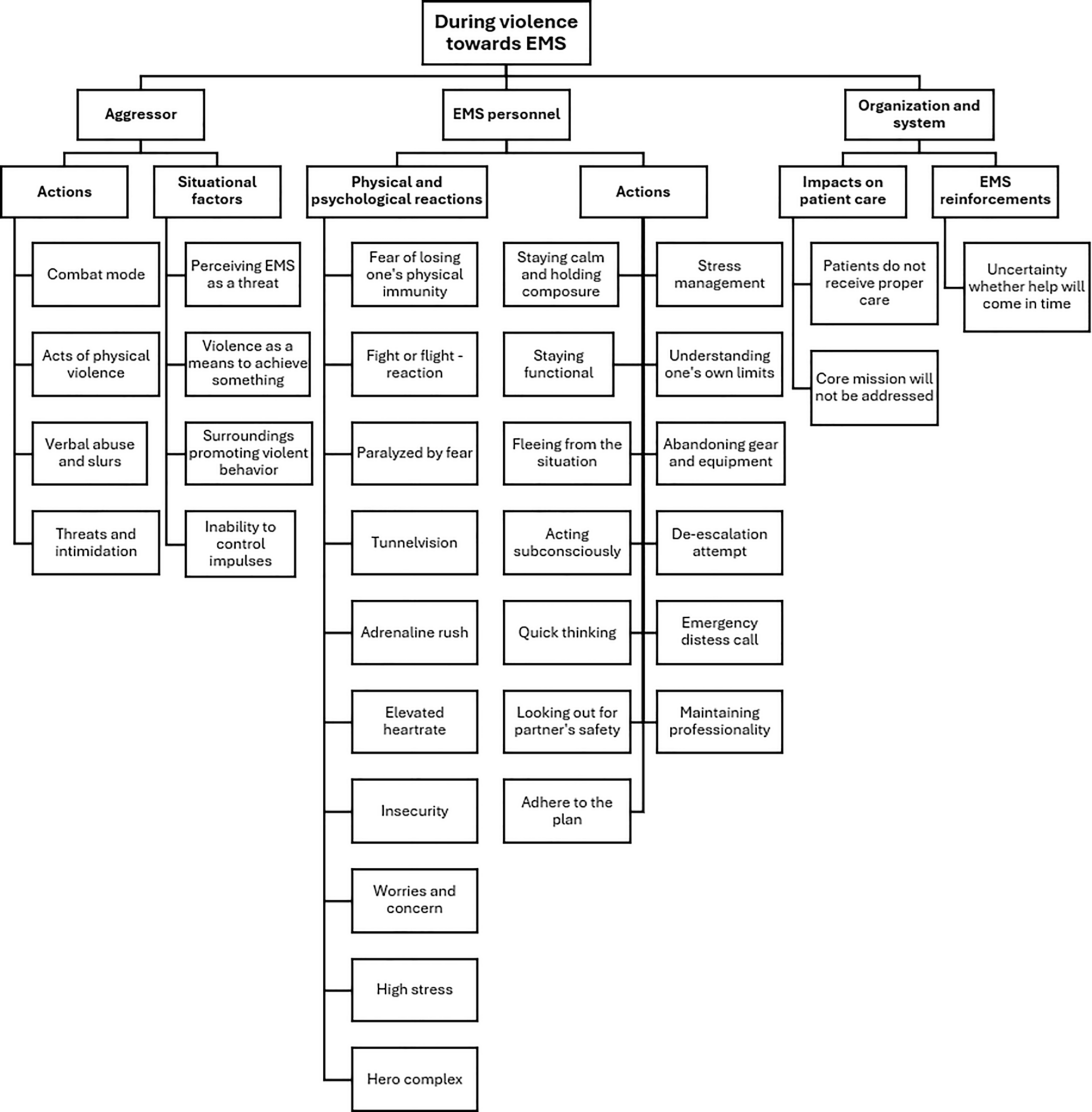
Identified factors during violence towards EMS personnel

During the violence (Fig. 3.), two aggressor-related subthemes were developed capturing the dynamics of actions taken by the aggressor as well as influencing situational factors. Aggressors were described as entering a “combat mode”, where any EMS action taken could trigger further hostility. Violence done by the aggressor ranged from verbal abuse, including insults, threats or slurs, to physical assaults such as hitting, kicking, biting or strangling. Situational factors were described as intensifying the aggression: Perceiving EMS as a threat, using violence to obtain drugs or leverage treatment decisions, or being in chaotic environments with intoxicated bystanders. In many cases, poor impulse control and an inability to control oneself were seen as contributing to violent behavior.

Nowadays people have a lot of knives, guns and other weapons. During violence there is a concern whether one might be used. -Workshop 1, group 1a.

Patient enters ‘combat mode’ and patients in emotional turmoil cannot think with reason. -Workshop 2, group 2a.

For EMS personnel, two subthemes were developed during violent incidents capturing physical and psychological responses and the actions taken. Fear and loss of a sense of safety were dominant in participants’ responses, with many describing fight-or-flight reactions such as tunnel vision, racing heartbeat or an adrenaline rush. Some participants raised concerns that EMS personnel may act with a “hero complex,” leading some individuals to take unnecessary risks. Reported actions ranged from activating an emergency distress call (via a portable radio) and fleeing the scene to attempts at de-escalation through professionalism, calm dialogue with the aggressor and other de-escalatory methods aimed at reducing aggression. Participants emphasized the importance of composure: Avoiding provocation, managing stress reactions, adhering to any possible emergency plans and safeguarding one’s EMS partner was seen as crucial.

must know one’s own limits and capabilities in the ongoing situation. One should not sacrifice oneself. -Workshop 1, group 2a.

Stress arises, fear, anxiety, primitive reactions…the psychological load is immense. -Workshop 1, group 2a.

Two organizational and systemic subthemes during violent incidents were developed capturing EMS reinforcement and impact on patient care. Participants expressed concern about the reliability of emergency distress calls (via portable radio), whether due to long police response times, technical issues or uncertainty about whether the signal was sent or received. Any possible problems were seen as a heightened risk of being harmed by the aggressor. Violence was also seen as problematic due to it disrupting the mission itself, causing patient care to be delayed or disregarded depending on the severity of the incident.

Calling for additional help during (violent) encounter is not trained enough. -Workshop 2, group 2b.

Due to the threat of violence, work (patient care) doesn’t not get done or it gets done poorly. -Workshop 2, group 2a.

### After the violent incident

**Fig. 4:**
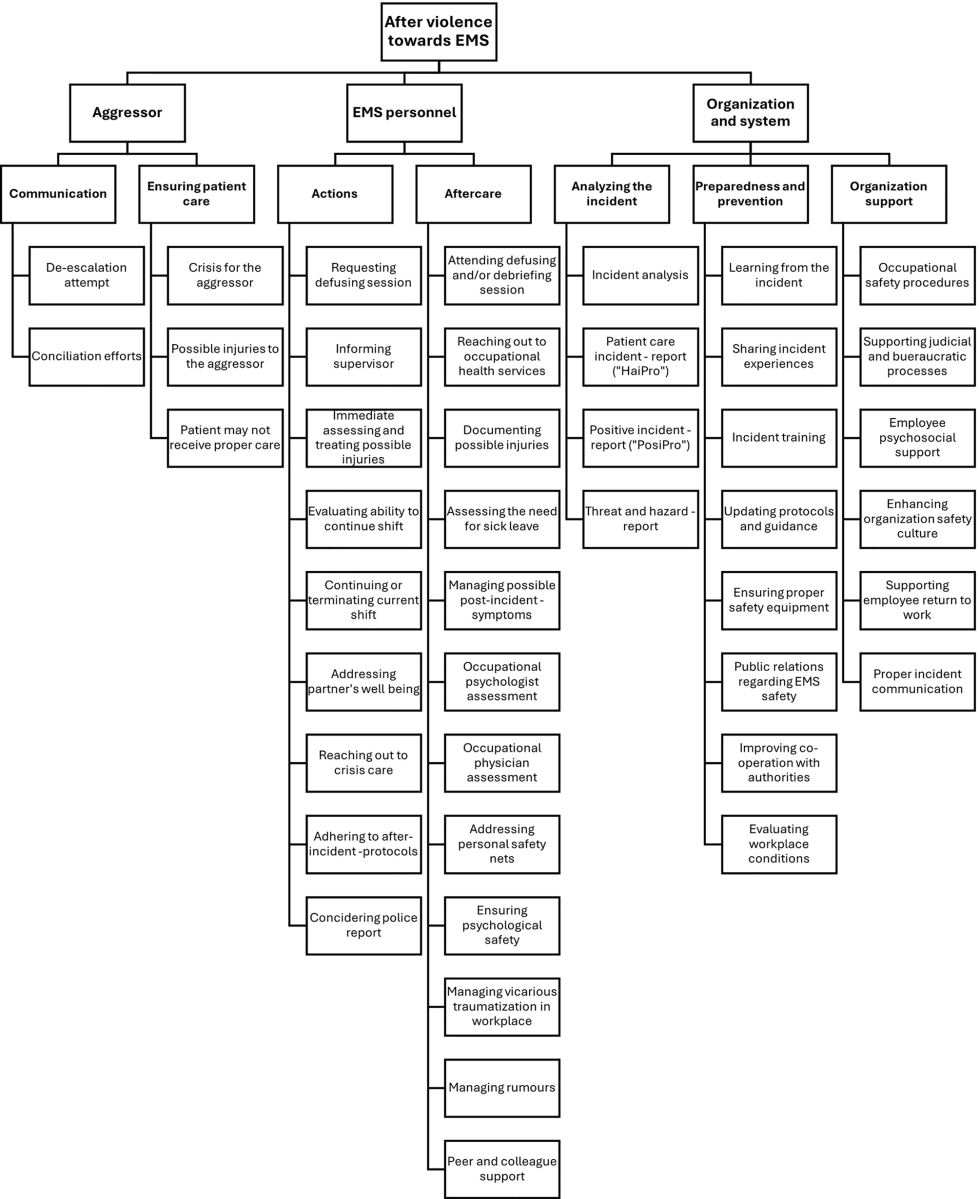
Identified factors after violence towards EMS personnel

After the violence (Fig. 4.), issues identified by the participants were happening either immediately after the violence, while the patient encounter was still ongoing, or clearly after the EMS mission had concluded. This time frame could be even days after the incident. The analysis developed two aggressor-related subthemes capturing communication and ensuring patient care. Participants emphasized the need for continued de-escalation so the mission could proceed safely, while also critically questioning how care could be managed when the patient was the aggressor. In such cases, police custody or use of force could delay or prevent treatment. Even self-inflicted or restraint-related injuries may risk being overlooked. It was also noted that violent incidents can potentially be traumatic for the EMS personnel as well as for the aggressive patient, raising the question of possible conciliation efforts to address the incident.

Crisis-situation for all parties. -Workshop 2, group 2b.

The other party (the aggressor) should be heard and given a possibility on conciliation. -Workshop 2, group 3a.

For EMS personnel, two subthemes were developed capturing immediate actions taken and aftercare. Immediately after an incident, staff were expected to inform supervisors, follow reporting protocols, assess, document and treat injuries and ensure partner wellbeing. Defusing sessions and crisis support were emphasized as crucial for deciding whether to continue or end the shift. Longer-term aftercare included occupational health assessments and safeguards for psychological safety, such as debriefing and similar sessions. Peer and colleague support was seen as ambivalent: While sharing experiences could aid recovery, it could also be problematic if it led to gossip or secondary traumatization within the working community

Can one keep continue working after a violent incident? -Workshop1, group 3a.

A debriefing session should be held immediately. All parties involved should attend. -Workshop 2, group 3a.

For organization and system, three subthemes were developed capturing analyzing the incident, preparedness and prevention as well as organization support. Reporting systems such as patient safety, hazards and “positive incident reports” were seen as essential tools for analyzing what happened, why and under which circumstances. Positive reporting was highlighted to share effective practices from challenging encounters. To prevent violent encounters, it was emphasized that organizations must learn from the incidents, update protocols, ensure training and strengthen cooperation with police. Necessary safety equipment was also seen as pivotal. Participants also highlighted the importance of employers guiding EMS personnel through occupational health, legal and bureaucratic processes, facilitating return to work and fostering a work culture where incidents can be openly discussed and learned from.

Employer should follow up on the employee… ask how they are doing and forward to professional help if needed. -Workshop 1, group 3a.

Analyzing the incident and taking proper action to prevent further incidents is employer responsibility. -Workshop 1, group 3a.

## Discussion

The phenomenon of violence towards EMS personnel appeared as a process involving the aggressor, the EMS personnel, and the organizational and systemic context. Together, these themes illustrate how violence is not a single event, but a dynamic interaction shaped by individual behavior, professional practice, and institutional conditions. These results help us better understand and design possible interventions for the management of violence risk throughout the entire EMS mission process.

Prehospital emergency care is inherently unpredictable, and EMS personnel have limited control over the individuals they encounter. Multiple factors were identified regarding the aggressor’s background and circumstances. Factors such as substance abuse, psychiatric illnesses and other mental health issues have been highlighted in previous studies [1, 6]. However, it is worth noting that although some patient groups may statistically pose a higher risk to EMS safety, categorically denying EMS response to these patients is not practical or ethically justified. This presents a difficult dilemma for EMS. Violent incidents can have direct consequences on patient care in the EMS setting. The nature and severity of violence determine the impact it might have. If the patient is the aggressor, the situation becomes more complex: Legislation and professional ethics obligate health care providers to treat all patients in need of care, making non-engagement problematic, even if the patient is violent. This poses a dilemma, balancing the patient’s right to care versus the EMS personnel’s right to personal safety. At this moment, no clear protocol addressing this dilemma has been issued. Further research and assessment are needed to determine the implications for how to develop the legislature and healthcare setting.

Challenges with EMS and police cooperation have previously been reported, leading to a sense of insecurity among EMS personnel [19]. In Finland, the police can offer aid to EMS in different scenarios according to the law [20, 21]. The core challenge is not with known threats where police involvement can be planned beforehand, but with sudden, unexpected or unrecognized violence during routine encounters. Potential predictive models to predict violent encounters in the EMS setting are lacking. So far, to the best of our knowledge, only one study has directly addressed this issue [5]. While having promising predictive value, further validation and practical integration research are still needed.

Based on our findings, the challenges in organizational guidance influence the risk of violence in EMS missions. Lack of tools to assess and anticipate violence risk in upcoming missions necessitates that EMS organizations prepare their staff for uncertain and potentially dangerous situations. Organizational and system protocols should clearly define thresholds for risk, specifying when an EMS unit should not engage alone and when police or other support must be involved [9]. Clear articulation of the threat assessment and shared understanding among EMS, police and other stakeholders are essential for safe practices. Best to our knowledge, no such protocols currently exist in Finnish EMS.

Study findings emphasize that the actions of the EMS personnel can influence the likelihood of violence. Behavior perceived as moralizing or condescending may provoke aggression, particularly in mentally unwell or intoxicated patients or among hostile bystanders. It is important to note that negative attitudes and stigmatization towards mental health patients may be present in EMS personnel [22, 23]. The personal characteristics of EMS personnel may also influence exposure to violence. Fatigue, poor stress tolerance and inexperience can increase vulnerability, while emotional resilience and strong situational awareness may be protective factors. Patients may instinctively interpret EMS personnel’s verbal and non-verbal behavior, and while it often has no consequences, it can sometimes provoke hostility, especially in patients under great stress or with mental health problems [24].

The perceptions of violence differ significantly among EMS personnel. What one professional considers violence; another might view as an unavoidable aspect of the job. Desensitization, normalization of violence and feelings of futility can lead to underreporting [1, 3, 7, 25]. While unprovoked violence most certainly sometimes happens in healthcare settings, it is also important to assess whether the failure to recognize potential warning signs contributed to the violence. The uncertainty of the EMS setting complicates the development of standardized protocols for preventing and responding to violent encounters. It also highlights the need for nuanced after-care systems that recognize subjective experiences of distress, even in the absence of physical harm. Organizations need clear criteria for when to initiate post-incident support, based not only on objective risk but also on individual experiences of how the incident was perceived by the EMS personnel involved. The recognized issue of underreporting should also be addressed at the organizational level. Reversing underreporting can be achieved by strengthening organizational culture and processes [25].

Another underexplored aspect in contemporary research is the aggressor’s experience. Participants highlighted that the violent incident affects both the aggressive patient and the EMS personnel. It was characterized as a crisis for the EMS as well as for the aggressor. Despite this, there is limited evidence about the aggressor’s perspective regarding violent incidents. One study analyzed court transcripts of offender experiences, highlighting the importance of communication skills among EMS personnel [26]. It should be acknowledged that acting violently is a dynamic process between the EMS personnel and the patient where individual decisions, behavior, communication and cognitive capabilities influence each other [24]. Currently, there are limited procedures for the patient to reconcile with the EMS victim regarding the incident. Exploring such victim-offender mediation could be valuable in long-term violence prevention strategies [27].

This study also offers more insight into pre-incident indicators, such as perceived behavioral cues and circumstantial conditions that may precede aggression. Multiple factors have been associated with higher risk of EMS violence in previous studies. EMS training and protocols, including escape protocols or targeted use of de-escalation tools, could evolve toward proactive prevention, if reliable predictors can be identified in real-time. In Finland, de-escalation and self-defense training is a part of the EMS education, but further development and standardization are warranted. Situational awareness and decision-making training focusing on violent encounters seems to be infrequent in EMS education in Finland [28]. Organizational factors play a dual role: they are pivotal both in preventing violence and responding to it. During severe violent events, participants viewed EMS system support as limited, with police or backup EMS units often being the only available assistance. Even then, the fastest assistance response times are measured in minutes, during which the EMS personnel may be at significant risk if the violence persists. Hence, the emphasis should shift toward violence prevention. The need for personnel training, strengthening organizational support and aftercare, and improving collaboration with other stakeholders has also been recognized in other studies [19, 29].

It is crucial to understand the importance of the need for further research and developing preventive measures for preventing and managing violence in EMS [30]. After a violent incident, the organization should systematically take responsibility for an incident analysis and proper response to prevent future incidents. Additionally, reversing the normalization of violence and underreporting will require systemic change in the EMS culture regarding how violent encounters are valued, the sustained organizational commitment and the inter-agency collaboration. Organizations should adopt a more proactive and granular reporting method than traditional retrospective surveys and other methods currently used to enhance reporting and incident analysis after violent encounters [31].

## Limitations

The workshop method, while encouraging collaboration and discussion, may cause some participants to moderate their narratives due to possible social pressure or hierarchical dynamics among the participants. Such dominance effects may influence data production in group-based qualitative research. Data were also based on participant-generated notes rather than recorded transcripts. While this approach reduces researcher interpretation during data capture, it may also cause selective recall as well as varying levels of detail in documenting. Participants continued discussing and documenting until data saturation was achieved. Sample size (*n* = 36) was considered adequate for this type of qualitative study. Rich and diverse data across relevant professional groups was collected, and thematic saturation was achieved. The strength of this study was the interprofessional approach, and it provided additional insight and depth by including patient participation. However, while the participants in the workshops were carefully chosen to represent relevant backgrounds, it is still possible that some perspectives or viewpoints were overlooked. The risk of selection bias was mitigated by recruiting people covering key roles in EMS and its relevant fields. However, some degree of selection bias remains possible due to the voluntary nature of participation. Possible unknown biases among participants might have also been present during workshops.

## Conclusion

This study reinforces the idea that violence against EMS personnel is a multifactorial phenomenon shaped by aggressor behavior, EMS personnel’s attributes and actions as well as the EMS system and organization level factors. It highlights that violence is a dynamic interaction involving both the aggressor and the EMS personnel. EMS system presents itself more as a facilitating and supporting entity, while the EMS personnel work directly and independently with the patient. This highlights the importance of EMS personnel also reflecting their own actions during EMS missions to minimize the risk of provocation and putting themselves in the harm’s way. Organizations must strengthen the EMS system with clearer risk assessment protocols, dynamic decision-making thresholds and stronger cooperation with law enforcement. The three-theme model identified in this study is consistent with process-oriented safety approaches. Structured risk-management methods, such as Failure Mode and Effects Analysis (FMEA), could help EMS organizations systematically address safety weaknesses across the phases before, during, and after a violent incident. Such approaches may also strengthen organizational safety culture and support leadership development.

Systematically embedding de-escalation, communication and disengagement strategies into continuing professional development could also enhance EMS preparedness. The absence of structured post-incident care processes, including psychological support for the EMS personnel, highlights a significant gap. Exploring victim-offender mediation may also offer new opportunities for recovery and long-term violence prevention.

## Supplementary Information

Below is the link to the electronic supplementary material.

Supplementary Material 1

## Data Availability

Due to participants’ privacy, the data is not publicly available.

## Abbreviations

EMS: Emergency medical services
ALS: Advanced life support
BLS: Basic life support
ERC: Emergency response centre
NEWS: National early warning score
